# The Promising Role of Plant-Derived Lectins in Oral Cancer Therapeutics: A Systematic Review

**DOI:** 10.7759/cureus.75910

**Published:** 2024-12-17

**Authors:** Saranya Ramsridhar, Chandini Rajkumar, Murali Balasubramaniam, Soumya Anandan, Mythili Sabesan, Logeswari Jayamani

**Affiliations:** 1 Department of Oral Pathology, Sathyabama Dental College and Hospital, Chennai, IND; 2 Department of Oral Pathology and Microbiology, Sri Ramachandra Dental College, Sri Ramachandra Institute of Higher Education and Research, Chennai, IND; 3 Department of Oral Pathology, Meenakshi Ammal Dental College, Chennai, IND

**Keywords:** apoptosis, autophagy, oral cancer, plant lectins, signalling pathways

## Abstract

Oral cancer (OC) continues to pose a significant global health challenge, marked by high morbidity and mortality rates despite advances in diagnosis and treatment. Numerous novel potential anticancer drugs have been evaluated, many of which are derived from natural sources, such as microorganisms, plants, and animals. Among these, plant lectins - a distinctive group of proteins and glycoproteins with strong biological activity - have garnered considerable attention over the years. Several plant lectins can trigger selective apoptotic cancer cell death or possess antiproliferative properties. The objective of this systematic review was to provide insight into the potential applications of plant lectins in the treatment of OC. Plant lectins suppress cancer cells by inducing apoptosis and/or autophagy by modulating various signalling pathways such as the caspase family, mitochondrial-mediated ROS-p38-p53 pathway, PI3K/Akt, and Wnt/β-catenin to inhibit OC. Multiple lectins have been shown to exhibit anticancer properties in cell cultures and in vivo. *Polygonatum cyrtonema* lectin, *Maackia amurensis* seed lectin, abrus agglutinin, wheat germ agglutinin, mistletoe lectin, and concanavalin A are among the plant lectins with the highest potential for anticancer activities. This review provides an overview of the current understanding of the role of lectins in cancer diagnosis and therapy, highlighting their potential applications and underlying mechanisms.

## Introduction and background

Oral cancer (OC) is a worldwide threat with around 2,75,000 cases newly diagnosed and 1,28,000 fatalities annually. Over 90% of the OCs are oral squamous cell carcinomas (OSCC), which develop in the mucous membranes of the oral cavity/oropharynx [1]. The worldwide prevalence of cancer differs significantly by geographical area, with developing nations accounting for over half of the cases worldwide [2]. Around 30% of all new cases of OC occur in India each year, making it one of the countries with the highest rate of occurrence [3]. Individuals with advanced OSCC have a five-year survival rate of 63% owing to resistance to therapy and low effectiveness of second-line chemotherapy [4]. As a result, novel approaches to therapy must be devised to reduce drug resistance and enhance the effectiveness of treatment. Several investigations have looked into the probable mechanisms behind medication resistance in OSCC, and many associated signalling pathways have been documented, notably Akt and MEK/ERK1/2 signaling [5,6]. Consequently, a high-potency medication with selective targeting choice and strength is required for cancer diagnosis, treatment, and mitigation [7].

Phytochemicals are gaining popularity in cancer treatment because of their low cost, accessibility, and low toxicity compared to traditional chemotherapies [8]. The universe of plants constitutes one of the foremost potential arenas to uncover and develop novel medications. Although complementary therapy, including traditional remedies, has lacked tangible evidence to substantiate its use in everyday life for centuries, numerous plant-based secondary metabolites are now being commercialized for the management of specific ailments [7,9].

Lectins are non-immunogenic molecules that bind specifically to the carbohydrate component of glycogen conjugates [10]. Lectin refers to a class of plant-derived agglutinins that agglutinate the red blood cells and are noted for their strong sugar-binding, biotechnological, and medicinal activities [11]. The particular molecular locations on lectin engage with mono- or oligosaccharides using non-covalent factors such as van der Waals, hydrophobic binding, and hydrogen bonds, with great affinities and specificity without negligible catalytic or immunological reactions. Despite being found in animal species, fungi, and bacteria, plant-based lectins have anti-inflammatory, anticancer, antimicrobial, immune-modulating, and antifungal properties. They could also be used as histochemical markers, drug delivery intermediaries, or disease biosensors [12,13].

Plant lectins may induce cell death in cancerous cells by generating abnormal glycan complexes on their membranes. They detect and address such alterations in malignant cells, demonstrating their effectiveness in cancer detection and therapy. The capacity of lectins to specifically target a variety of glycosylated biological elements on the cell surface has transformed their use in pharmaceutical research, particularly in the treatment of complex disorders [14,15]. Previous research has shown that several plant lectins can trigger selective apoptotic cancer cell death or function as antiproliferative entities [16]. The objective of this systematic review was to provide insight into the potential applications of plant lectins in the treatment of OC.

## Review

Materials and methods

Study Protocol

The systematic review adhered to the Preferred Reporting Items for Systematic Reviews and Meta-Analyses (PRISMA) standards for research selection, synthesis, and reporting [17]. The focused research question was "What are the potential roles of plant lectins in the management of oral cancer?"

Information Sources and Search Strategy

The PubMed, Scopus, and Cochrane databases were searched for evidence-based research papers on the potential effects of plant-based lectins on OSCC published between January 2004 and July 2024. After reviewing the literature, the following Medical Subject Headings (MeSH) phrase combination was used: (“Plant-derived lectin” OR “Agglutinin” OR “Mistletoe lectin” OR “Abrus agglutinin” OR “Wheat germ agglutinin” OR “Viscum album L. var. coloratum” OR “Maackiaamurensis seed lectin” OR "Concanavalin A" OR “Polygonatumcyrtonema lectin”) AND ("Chemoprevention" OR "Cell Culture" OR "Cell lines") AND ("Oral Squamous Cell Carcinoma" OR "Head and Neck Squamous Cell Carcinoma" OR "Oropharyngeal Carcinoma" OR “Oral Cancer”) AND ("Apoptosis" OR "Proapoptosis" OR “Autophagy” OR "Cytotoxicity" OR "Interleukin" OR "Signalling Pathway" OR "MEK/ERK1/2 signalling"). The citations of each listed article were manually reviewed to find studies not in the web-based databases. Two independent experts performed an independent search and predefined screening. If two reviewers disagreed, a third reviewer made the ultimate judgment at each stage of the review. The three reviewers agreed unanimously.

Eligibility Criteria, Study Population, Design, Intervention, Comparison, and Outcome

This systematic review applied Population, Intervention, Comparison, Outcome, and Study Design (PICOS) criteria. The population includes individuals with OSCC or any other cancers associated with the oral cavity who are 18 years old and older, as well as OC cell lines and animal models used in OC research. The study design includes in vitro, animal, or clinical trials to evaluate the role of plant-derived lectins in OC. The intervention involves research on the potential application of plant-derived lectins in the treatment of OC. The comparison includes a placebo control group and the delivery of other synthetic chemotherapies. This study assessed the potential effects of plant-derived lectins on OC by evaluating cellular proliferation, cytotoxicity, apoptosis, cell cycle arrest, protein expression, and chemopreventive or therapeutic effects through in vitro and in vivo experiments.

Systematic reviews, questionnaire surveys, editorial comments, case reports, case series, and pilot studies were not considered. Articles not written in English were excluded as this can lead to biased results and reduced generalizability. Non-English articles lead to professional translation services that can be expensive and time-consuming and can increase resource challenges as well. Articles not focusing on the antitumor action of plant lectins and studies investigating various cancers other than those associated with the oral cavity were also eliminated.

Study Selection

Both reviewers separately evaluated the titles and abstracts of the included articles. Papers that failed to satisfy the requirements for consideration were not examined. Reviewers independently assessed the selected full-text articles. The selected studies' citation lists were searched for related papers.

Data Extraction

Data were collected using a predetermined format that comprised the first author's name, publication year, study location, study design, cell lines/animal models utilized, and signalling pathways of lectin. The corresponding author was contacted for additional information when required.

Quality Assessment

The Cochrane Collaboration tool measured the risk of bias (RoB) in non-randomized trials (ROBINS-I) as low, moderate, serious, critical, or no information [18]. The tool includes confounding bias, participant selection bias, intervention bias, deviation from targeted intervention bias, missing data bias, outcome assessment bias, and selective reporting bias. Overall RoB for the various investigations was categorized as follows: low RoB was given when all requirements were met. A moderate RoB was assigned to studies that did not meet any domains. A significant RoB occurred when at least one domain expressed serious concerns on any given domain. Critical concerns in any one area indicate a critical RoB for the investigation. A study with no information and no apparent evidence of serious or critical RoB was awarded no information.

Results

**Figure 1:**
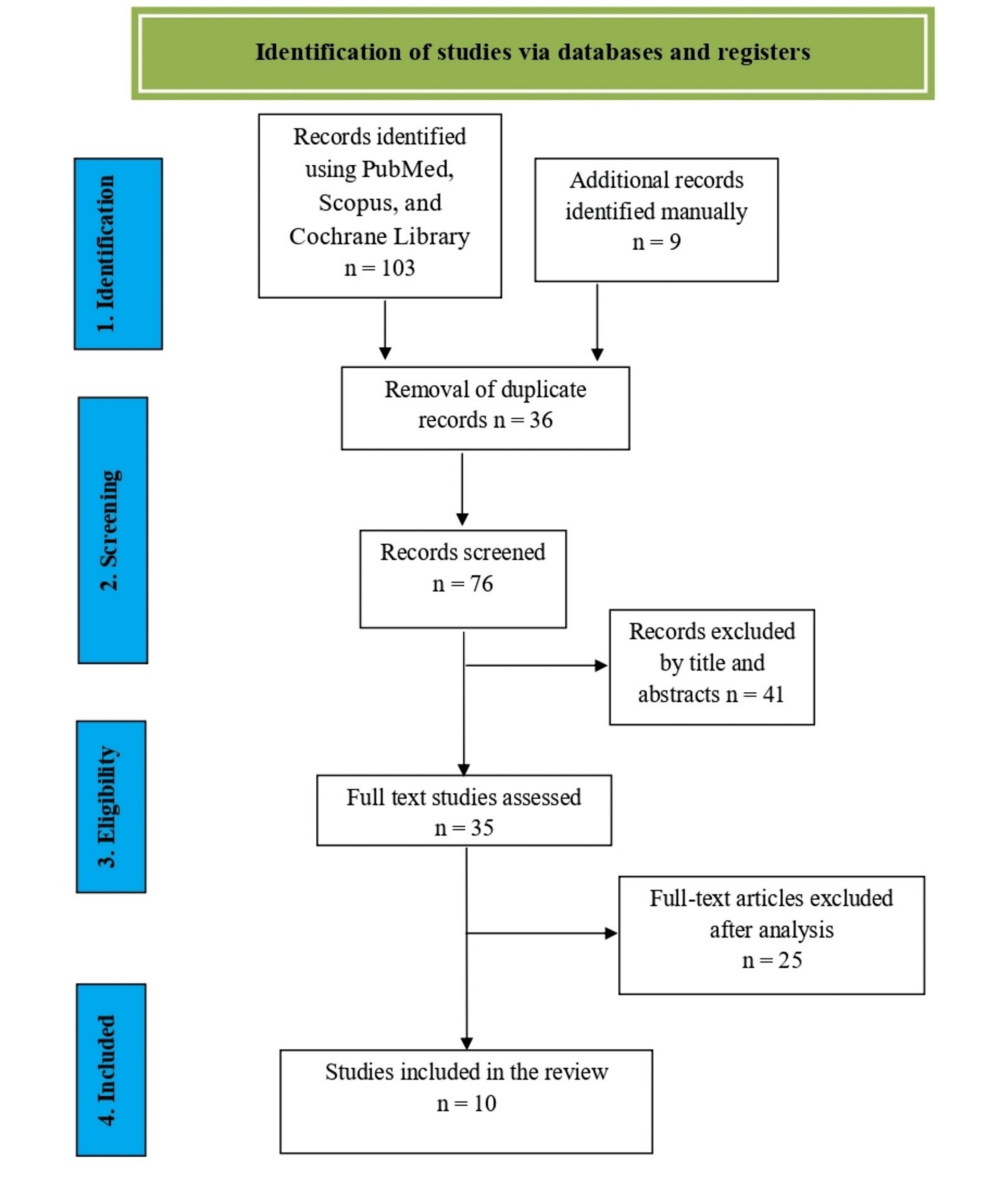
PRISMA flow chart of the reviewed studies. PRISMA: Preferred Reporting Items for Systematic Reviews and Meta-Analyses

Figure 1 demonstrates the progressive process of study selection. Following an examination of the databases listed above and a manual search, 112 studies that met the inclusion criteria were selected. There were 36 duplicate items deleted, yielding 76 publications for title and abstract evaluation. The review invalidated 41 articles. In total, 35 full-text publications were reviewed. Following full-text screening, 25 publications were removed as their content did not meet the established standards. The literature search was completed in less than two weeks, and finally, 10 studies were included in the present review (Table 1) [19-28].

**Table 1:** Characteristics of the reviewed studies. MASL: *Maackia amurensis* seed lectin; WGA: wheat germ agglutinin; ROS: reactive oxygen species; AGG: Abrus agglutinin; PCL: *Polygonatum cyrtonema *lectin; VCA: *Viscum album* var. coloratum agglutinin

Studies	Country	Study design	Lectin extract type	Cell lines/cancer types	Mechanism of cell growth inhibition	p-Value
Hamilton et al. (2021) [19]	USA	In vitro	MASL	HSC-2 cells of OSCC	MASL suppresses the expression of JAK-STAT, TGFβ-SMAD, and Wnt-βCTN signalling pathways, hence restraining the development and migration of OSCC.	<0.0001
Silveyra et al. (2021) [20]	Uruguay	In vitro	WGA	Normal oral mucosa (n=3) and OSCC (n=5)	The staining pattern of WGA indicates the level of cell cohesion in OSCC. This cohesiveness reduces in the basal layers and invasive tumor clusters, which exhibit more migrating topologies. Combining WGA staining with confocal microscopy might serve as a beneficial method for investigating the structure of tissues in OC.	NA
Panigrahi et al. (2020) [21]	India	Animal study (male Syrian hamsters)	AGG	CAL33, CAL27, 22A, 22B, FaDu, and normal keratinocytes HaCaT cells	AGG reduces the levels of NRF2 by breaking down p62 through autophagy, leading to the stimulation of ROS and the promotion of cell death. This property could be harnessed for the development of a natural anticancer medication to combat the spread of OC.	NA
Sinha et al. (2017) [22]	India	In vitro and in vivo using xenograft mouse model	AGG	FaDu, SSC4, and SSC25 cells	AGG shows potential as a viable alternative to conventional therapy for oral cancer by specifically affecting the Wnt/β-catenin pathway and inhibiting antiapoptotic signalling in the oral microenvironment.	<0.05, <0.01
Sinha et al. (2017) [23]	India	In vitro and in vivo using xenograft mouse model	AGG	FaDu, HEp-2, RPMI2650, SCC-4, SCC-9, SSC-15, and SSC-25	The expression of PUMA was increased in a fashion that depended on the dosage of AGG, indicating that the induction of p73 by AGG triggered apoptosis mediated by PUMA. Ultimately, we have determined that there is a relationship mediated by AGG between ROS, DNA damage, ATM, and p73 in FaDu cells. This link may have a substantial impact on enhancing the treatment potential for p53-deficient OSCC.	<0.05
Han et al. (2015) [24]	Korea	In vitro and in vivo	Korean mistletoe coated with a biodegradable polymer (EudragitÒ)	B16BL6 and B16F10 (mouse melanoma) cells	The combination of VCA with mistletoe extract resulted in a potent G0/G1 cell cycle arrest and apoptosis in B16BL6 and B16F10 cells. Furthermore, a decrease in the levels of procaspase-3 and -8 expression, together with an increase in the activation of several caspases, were identified. This observation may indicate a potential mode of action that might indicate apoptosis.	>0.05
Ochoa-Alvarez et al. (2015) [25]	USA	In vivo	MASL	HSC-2, HSC-4, and HSQ-89 cells (OSCC)	Monoclonal antibodies (NZ-1) and lectins can be employed for combating OSCC and other malignancies that exhibit podoplanin.	<0.001
Liu et al. (2009) [26]	China	In vitro	PCL	A375 cells	PCL causes mitochondrial ROS-p38-p53-mediated apoptosis and autophagy.	<0.01
Liu et al., 2009 [27]	China	In vitro	Concanavalin A	A375 cells	Concanavalin A causes caspase-dependent and mitochondrial apoptosis in A375 cells.	<0.05
Choi et al. (2004) [28]	Korea	In vitro	VCA (mistletoe lectin)	Human A253 cancer cell line	VCA regulates Akt/PKB signalling pathways in A253 cells, potentially leading to telomerase suppression and apoptosis. This suggests that VCA could serve as an effective chemotherapeutic treatment for OC cells.	NA

Because of their ubiquitous prevalence and comparable protective qualities, plant lectins have received the most attention in lectin research. *Polygonatum cyrtonema* lectin (PCL), *Maackia amurensis* seed lectin (MASL), Abrus agglutinin (AGG), wheat germ agglutinin (WGA), mistletoe lectin, and concanavalin A (ConA) are among the plant lectins with the highest potential for anticancer activities. Plant lectins modulate signalling pathways, such as the caspase family, mitochondrial-mediated ROS-p38-p53 pathway, PI3K/Akt, and Wnt/β-catenin, to inhibit OC. MASL affects genes involved in the TGFβ-SMAD, JAK-STAT, and Wnt-βCTN signalling cascades. MASL specifically reduced PDPN, SOX2, SMAD5 RNA, protein expression and also decreased SMAD and MAPK action, indicating the likelihood of combined treatment with doxorubicin and 5-fluorouracil. HSC-2 cells treated with 0, 0.770, or 1.925 nM MASL migrated an average of 343±30, 115±16, and 44±5 µm with n=3, where 0.770 nM MASL for 6 h showed a high significance with p<0.0001 [19]. In *Viscum album* var. coloratum (VCA)-treated A253 cells, caspase-3 activation induced apoptotic cell death, while transcriptional down-regulation of hTERT inhibited the action of telomerase. It was also shown that diminished phosphorylation of the Akt survival signalling pathway inhibited the actions of telomerase and induced apoptosis. With the incorporation of VCA, the degree of Akt/PKB phosphorylation decreased substantially in A253 cells [28].

Silveyra et al. utilized fluorescently labeled WGA, tyramide signal amplification labeling of structural proteins, and confocal microscopy to visualize the tissue structure in OSCC thin slices preserved in formalin and embedded in paraffin. WGA staining defined cell borders strongly in the exterior layers, but appeared diffuse in the basal layers, indicating a shift from periphery to cytoplasm in the arrangement of structural proteins E-cadherin, β-actin, and syndecan-1. WGA labeling allows for a clear distinction between tumor cell clusters and the adjacent stroma in tumor infiltration locations. Tumor cell clusters with diffused WGA staining exhibited cytoplasmic redistribution of E-cadherin, β-actin, syndecan-1, and morphological alterations from polygonal to elongated cells [20].

Tumor cell clusters with well-defined WGA staining at cell boundaries were observed whereas other clusters showed a much more diffuse WGA staining pattern, marked by prominent cytoplasmic E-cadherin, β-actin, and syndecan-1 staining [20]. In a study by Sinha et al., the OSCC was treated with different concentrations of AGG (0.1, 0.5, and 1 μg/mL). After 24 h treatment, the CD44-cell populations increased from 27±4.1 in control to 58±6.2, 60±5.3, and 61±4.1 with a p<0.05. Whereas, SCC25 cells displayed 4±1 in control to 13±2.5, 17±1.5, and 18±1.0 in a dose-dependent manner with a p<0.01 [22]. In another study by Sinha et al., OSCC treated with AGG for 12 h showed a significant result in FaDu cell counts, with p<0.05 [23]. The effect of mistletoe granules with enteric coating on mice transplanted with melanoma cells (B16F10) was investigated by Han et al. In this study, the positive control group was treated with polysaccharide K (500 mg/kg) at concentrations of 1%, 2%, and 4%, while the negative control group was treated with 0.85% NaCl. The percentage of tumors was compared between these groups. After 14 days, positive control exhibited a reduction in tumor size but statistically showed a non-significant result with p>0.05 with one-way ANOVA [24].

Podoplanin expression was analyzed with OSCC cell motility by Ochoa-Alvarez et al., cell migration was evaluated by wound healing and quantitated as the number of cells that migrated into a 200x300 µm area in the center of the wound at 18 h (mean+SEM, n=5). When compared to HSC-2, HSC-4 showed a significant result with p<0.05 and HSQ-89 showed a p<0.001 [25]. In a study by Liu et al., upon treating A375 cells with 15 μg/mL *Polygonatum cyrtonema* (in the presence or absence of N-acetyl-cysteine), the flow cytometry showed an autophagic ratio with p<0.01 [26]. In another study by Liu et al., the antiproliferative effect of ConA on A375 human melanoma cells was assessed using the MTT assay. After treatment with 25 µg/mL ConA for 12, 24, or 36 h, significant results were observed (p<0.05) for apoptosis and necrosis at 35 h, with mean±SD values of 50±0.5 and 10±0.5, respectively [27]. In a study by Choi et al., the IC50 of VCA was measured in A253 cells after treatment for 24 h with VCA at the indicated doses. The mean±SD was found to be 10±2 ng/mL [28]. Six of the 10 studies showed a significant result on the efficacy of plant lectins in OSCC with p<0.05. In a study by Han et al., mistletoe did not produce a significant result in tumor reduction, with p>0.05 [24].

Quality Evaluation of the Studies Examined

**Figure 2:**
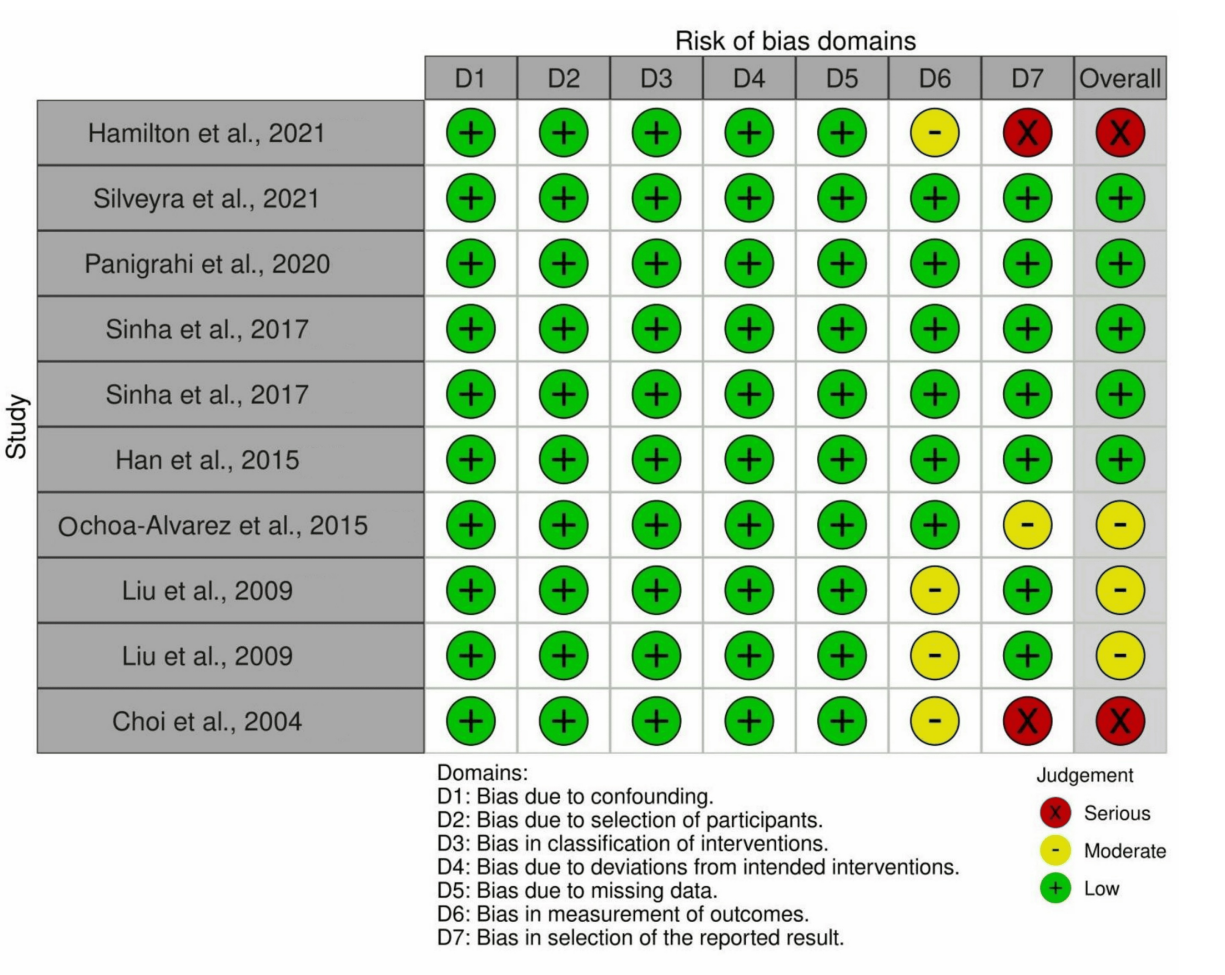
ROBINS-I tool for the RoB evaluation within the reviewed studies. ROBINS-I: Risk Of Bias In Non-randomized Studies of Interventions; RoB: Risk of Bias

**Figure 3:**
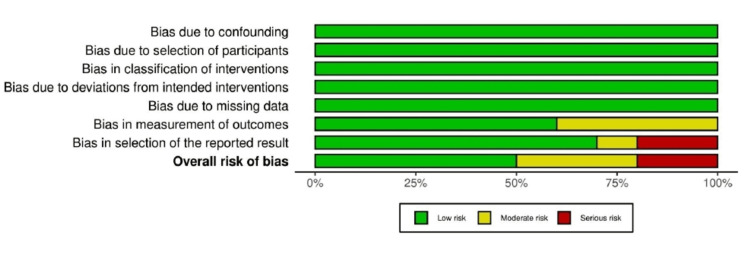
ROBINS-I tool for the RoB evaluation across the reviewed studies. ROBINS-I: Risk Of Bias In Non-randomized Studies of Interventions; RoB: Risk of Bias

Five studies analyzed had low RoB, as determined by the ROBINS-I approach (Figure 2) [19-28]. However, five other investigations stood out, with two demonstrating severe RoB [19,28] and the other three revealing moderate RoB [25-27]. Figure 3 shows the RoB across the studies.

Discussion

Developing new, powerful anticancer drugs may be aided by knowledge of the cytotoxic and antiproliferative actions of plant lectins on cancer cells. Previous studies have shown that plant lectins may adhere to tumor cell surfaces and induce apoptosis and autophagy. The lectins modulate apoptotic and autophagic signalling pathways to induce cell death. Plant lectins, due to their particular carbohydrate binding capabilities, have been widely used as probes to analyze the surface topography of normal and altered cells. Detecting variations in cell surface carbohydrate production with lectins has predictive value [29]. ConA lectin is a potent anticancer agent because it preferentially binds to malignant cells and deactivates many RTKs. Similarly, ConA can cause tumor cell death through coupling to MT1-MMP and RTK receptors, affecting downstream signalling pathways notably PI3K/Akt, JAK/STAT, MAPK, and NF-κB [30].

MASL targets human cell-expressed glycoproteins [31], prefers O-linked carbohydrate chains with sialic acid [32], and targets podoplanin to suppress tumor cell proliferation and migration at nanomolar doses [33]. WGA is a plant-derived lectin that binds to membrane glycoproteins expressing N-acetylglucosamine and sialic acid, particularly adhesion molecules found on cell membranes. In tumor invasion areas, WGA labeling distinguishes tumor cell clusters from stroma [20]. AGG, a plant lectin from *Abrus precatorius *seeds, specifically reduced cell proliferation and induced cell cycle arrest and mitochondrial death in FaDu cells via ROS-mediated ATM-p73 [23]. *V. album* formulations have been tested for anticancer activity alone or in combination with current therapies, but their precise mechanism of action and therapeutic advantages are unknown. A systematic review of clinical trials by Kienle and Kiene assessing mistletoe extracts found improved survival, no unfavorable interactions with the anticancer drugs being used, and a reduction in adverse events associated with conventional cancer therapy [34].

Another systematic evaluation reported by Ernst et al. consisted of 11 randomized clinical trials on mistletoe extracts, including uncontrolled, historically controlled, and retrospective studies. Only four of the 11 studies included in the review were adequately randomized. Both adjuvant and mono-therapy trials of mistletoe extracts were considered. In contrast to the present systematic review, this study did not lend strong evidence of the effectiveness of mistletoe extracts as a therapeutic agent for cancer. However, the study in itself reported a few limitations such as a high risk of bias among the included studies and the usage of several methods of extract preparations for different types of cancers [35].

Although the details of tests may differ, most researchers adhere to a common framework to evaluate the efficacy and processes of specific lectins on rapidly dividing cells. Before testing, it is necessary to purify any lectin from its source, similar to other biological materials. The procedure for this stage may differ between studies; however, it can be accomplished through salting out, dialysis, or a range of chromatography techniques such as gel filtration, ion exchange chromatography, affinity chromatography, and high-pressure liquid chromatography [36]. After chromatographic purification, gel electrophoresis is employed to segregate the different proteins and extract the individual proteins under study. A lectin gene can be introduced into cancerous cells by various methodologies. A widely employed technique in laboratories involves the use of a virus vector that carries the gene responsible for encoding a particular lectin. Gel blot hybridizations and transfections, a non-viral technique for introducing lectins, are employed to introduce lectin plasmids into cells, enabling the production of lectins [37,38].

In human melanoma A375 cells, PCL induces autophagy and death utilizing a mitochondria-mediated ROS-p38-p53 cascade [26]. Autophagy and apoptosis are implicated in another cancer therapeutic strategy. When cleaved, Atg5, an autophagic protein, activates apoptosis [39]. Autophagic processes may help determine neoplastic cell death strategies. Lectin chemotherapy effectiveness depends on apoptosis and autophagy. To help bridge the gap between research and practical application and to foster an in-depth understanding of the potential effects played by lectins in both autophagy and apoptosis, more funding should be allocated to such studies. In vitro laboratory and in vivo animal trials exhibit beneficial effects, but clinical studies are warranted to advance lectin-based chemotherapy for cancer. Plant extract with lectin has been employed therapeutically at low dosages to manage various tumors without major adverse effects and appears to be effective in certain circumstances [14].

Several issues with lectin-based cancer studies include manufacturing, non-specific binding, and low stability. *Cratylia mollis* (Cra) lectin liposomes were developed to solve these issues in lectin anticancer therapy. Cra-lectin, a mannose and glucose-binding lectin, was tested for anticancer activity in mice sarcoma cells. Cra-lectin nanoparticles had better tumor suppression, protein stability, and tissue cytotoxicity than the free solution [40]. Due to the ease of administering drugs and therapeutic effect analysis, lectin-conjugated nanoparticles have garnered recognition as drug delivery platforms. Leukemia has been observed to benefit from theranostic lectin application, which uses lectin-conjugated paclitaxel-loaded nanoparticles. A nanoparticle formulation of paclitaxel was more effective against myelogenous leukemia cells than the original application [41].

Plant-based treatments for cancer have faced both successes and failures. Resveratrol, found in red grape skin, induces apoptosis in tongue squamous cell carcinoma cells by reducing cell viability, promoting apoptotic processes, and suppressing cell migration through the mitochondrial pathway and transcription factors, which trigger the epithelial-to-mesenchymal transition. Curcumin, a polyphenol found in turmeric rhizomes, exhibits potential as a cancer therapy by targeting pathways such as NF-κB and MAPK. Its antioxidant, analgesic, anti-inflammatory, and anticarcinogenic properties further enhance its therapeutic promise. Quercetin, a polyphenol present in various plants and fruits, exhibits anticancer properties by inhibiting enzymes involved in carcinogen activation and interacting with cellular receptors and proteins. In vitro studies demonstrate that quercetin can induce cell death in oral cancer, suppress the NF-κB, MMP-2, and MMP-9 signaling pathways, and hinder cancer cell migration and invasion [42].

However, the use of herbal medicine can have several potential drawbacks, including direct toxic effects, harmful interactions with anticancer drugs, and increased chemosensitivity of cancer cells, necessitating a reduction in dose density [43]. Epigallocatechin-3-gallate (EGCG), the major constituent of green tea, interacted with sunitinib, a novel oral multitargeted tyrosine kinase inhibitor for patients with metastatic renal cell carcinoma and advanced gastrointestinal stromal tumor which has a good prospect for clinical application and is being investigated for the potential therapy of other tumors. EGCG interacts with sunitinib and reduces the bioavailability of sunitinib [44]. EGCG and other polyphenols also prevented tumor cell death induced by bortezomib (BZM), a proteasome inhibitor in clinical use for multiple myeloma. EGCG directly reacted with BZM and blocked its proteasome inhibitory function; as a consequence, BZM could not trigger endoplasmic reticulum stress or caspase-7 activation and did not induce tumor cell death [45]. Concomitant use of Chinese herbs like *Oldenlandia diffusa* and *Rehmannia glutinosa *could result in induction of CYP3A4, leading to a reduced efficacy of drugs that are CYP3A4 substrates and have a narrow therapeutic window [46].

Despite the anticancer properties of lectin, there are a few limitations impeding their potential in cancer therapeutics. Toxicity is one of the potential limitations of lectin. A study conducted by Leist and Wendel identified that Con A, which is the most studied plant lectin and a potent antineoplastic component induced liver failure in mouse models upon intravenous administration [47]. In addition, *Phaseolus vulgaris* lectins are known for their anticancer and anti-HIV properties and for preventing mucosal atrophy. Yet, it induces diarrhea, nausea, and vomiting upon oral treatment [48]. Another animal study conducted by Coelho et al. reported that plant (*Annona coriacea*) lectin has been identified to exhibit toxicity to *Anagasta kuehniella* by modifying the gut membrane environment and interfering with the digestive enzyme recycling mechanism [49]. Detailed analysis of lectin effectiveness on certain cancer cell lines and the associated pathways and processes will offer a platform for future researchers. Further study on lectins, particularly randomized controlled clinical trials, is essential to establish the highly specialized proteins that have the best chance of combating a variety of malignant cell lines that jeopardize the well-being and health of humanity [50].

## Conclusions

In conclusion, lectins sourced from plants and animals show great potential for future cancer therapies. Research to date has revealed significant anticancer properties in various lectins, as discussed in this article. Both apoptosis and autophagy are crucial in determining the success of lectin-based chemotherapy. Further funded research with a larger sample size in the future is necessary to explore how lectins influence these processes and to support the transition from experimental studies to clinical applications. Although in vitro and in vivo studies have yielded promising outcomes, clinical trials are essential for advancing the use of lectins in cancer treatment.
